# “HOW DOES THAT MAKE YOU FEEL?” USING NETWORK SCIENCE TO ESTIMATE AFFECT DYNAMICS ACROSS ADULTHOOD

**DOI:** 10.1093/geroni/igae098.1991

**Published:** 2024-12-31

**Authors:** Lucas Hamilton, Eric Allard

**Affiliations:** Augustana University, Sioux Falls, South Dakota, United States; Cleveland State University, Cleveland, Ohio, United States

## Abstract

Research on affect dynamics has dramatically changed in the past two decades. With the proliferation of ecological momentary assessment and online data collection, methodological and analytical approaches have greatly expanded. One approach is the estimation of emotion networks which can be done through a few lines of R code but requires a great deal of planning and interpretation by the researcher. Network estimation models individual emotions as distinct (i.e., sad or angry) and dimensionally dependent (i.e., sad and angry). This technique can test the independent influence of each emotion and unique associations between every pair of emotions. Critically, network structures (i.e., magnitude of co-fluctuation, independence of items) can be compared both within-individuals and between groups. Our previous findings demonstrate the feasibility and utility of estimating emotional reactivity across the lifespan. Using a video clip elicitor and a sample of 531 people (178 younger, 175 middle-aged, and 178 older adults), emotion networks were used to verify mean affective reactivity before and after the clip while also testing distinct co-occurrences (i.e., mixed emotions). To further demonstrate the usefulness of this approach, we have undertaken a database project that evaluates affective blending in response to 48 video clips. Mirroring the methodology from above, a sample of younger, middle-aged, and older adults rated their emotions before and after each video clip they watched. After summarizing overall reactivity findings, we will discuss how emotion networks offer great utility for disentangling unique and co-occurring emotions and their importance in emotional aging research.

